# Effects of maternal arginine supplementation during lactation on offspring growth and wound healing in an experimental rat model of intrauterine malnutrition

**DOI:** 10.1590/acb411626

**Published:** 2026-04-10

**Authors:** Ayrton Alves Aranha, Jorge Eduardo Fouto Matias, Djanira Aparecida da Luz Veronez, Lucia de Noronha, João Carlos Repka, Rafaella Toledo Aranha, Antônio Carlos Ligocki Campos

**Affiliations:** 1 Universidade Federal do Paraná – Postgraduate Program in Surgery – Curitiba (PR) – Brazil.; 2 Hospital Pequeno Príncipe – Department of Surgery – Curitiba (PR) – Brazil.; 3 Universidade Federal do Paraná – Department of Anatomy – Curitiba (PR) – Brazil.; 4 Pontifícia Universidade Católica do Paraná – Curitiba (PR) – Brazil.; 5 Hospital Angelina Caron – Campina Grande do Sul (PR) – Brazil.; 6 Universidade Positivo – School of Medicine – Curitiba (PR) – Brazil.

**Keywords:** Fetal Nutrition Disorders, Wound Healing, Arginine

## Abstract

**Purpose:**

To evaluate the effects of maternal arginine-enriched diet during lactation, following gestational undernutrition, on offspring growth and wound healing.

**Methods:**

Pregnant Wistar rats were divided into two groups during gestation: control (C; *ad libitum* diet) and undernourished (Und; 60% of control diet). After delivery, the undernourished mothers were reassigned to two subgroups: *ad libitum* + Impact^®^ (Arg) and *ad libitum* + Nutren^®^ (Hh). Evaluated parameters included offspring anthropometric measurements, serum albumin, tensile strength, and collagen morphometry at the surgical wound. Statistical analyses included analysis of variance (ANOVA) and Tukey’s, Student’s t-test, and Fisher’s test.

**Results:**

Maternal immunonutrition during lactation promoted recovery of offspring weight (C = 29.21 ± 2.86 g; Arg = 36.08 ± 5.38 g; *p* = 0.0009) and body length (C = 9.87 ± 0.32 cm; Arg = 10.91 ± 0.73 cm; *p* = 0.0008), shifting values from significantly lower at birth to higher than controls by weaning. Offspring healing parameters showed no significant differences compared with controls.

**Conclusion:**

An arginine-enriched diet provided to lactating rats malnourished during gestation significantly improved offspring anthropometric growth and supported wound healing following surgery at weaning.

## Introduction

Malnutrition accounts for approximately 2.2 million deaths among children under 5 years of age worldwide^1^. In 2020, the World Health Organization reported that 149 million children under 5 were stunted and 45 million were wasted^2^. Among critically ill children, the prevalence of malnutrition is estimated at 37.2%^3^. The concept of the “first 1,000 days” highlights gestation and lactation as critical windows for intervention in childhood malnutrition^4^. Evidence indicates that up to 62% of birth weight variability is attributable to intrauterine conditions^5^.

Since the 1990s, when Barker et al. first linked intrauterine malnutrition to increased cardiovascular risk in adulthood, the issue has been extensively studied^6-9^. Maternal dietary deficiencies have been associated with preterm labor and small-for-gestational-age infants. Both clinical and experimental studies of preconceptional and prenatal malnutrition have demonstrated deleterious effects on the development of multiple organs and systems^10-16^.

The relationship between malnutrition and impaired wound healing has been widely discussed. Collagen synthesis depends directly on the availability of macronutrients such as carbohydrates, lipids and proteins, as well as micronutrients, such as vitamins and trace elements^17-20^. Lipid deprivation compromises cell replication and inflammatory metabolism^21^.

Maternal supplementation to prevent fetal malformations and support development has proven effective, particularly with folic acid and vitamins A and D during gestation^22-25.^ Arginine supplementation during pregnancy has demonstrated positive effects in reversing intrauterine growth restriction^26-30^. Mateo et al. reported improved weight gain in piglets associated with maternal arginine supplementation during lactation^31^. Arginine is a non-essential amino acid that has several special properties, such as protein synthesis, nitric oxide production, and synthesis of collagen for wound healing.

The aims of this study were: to develop an experimental rodent model of maternal supplementation during lactation in rats undernourished during gestation, and to evaluate the effects of arginine supplementation on neonatal growth and abdominal wall healing.

## Methods

The experiment was conducted in accordance with the Canadian Council on Animal Care guidelines and was approved by the local Animal Use Committee (protocol no. 23075.082514/2011-68). Five male and 20 female Wistar rats (120–140 days old) were mated at a 4:1 ratio during the dark period in polypropylene cages (46 × 31 × 21 cm). Animals were housed under controlled conditions with constant temperature (22 ± 2°C) and a 12-hour light/dark cycle. The presence of spermatozoa in vaginal smears was used to confirm mating, designating day 1 of pregnancy.

After pregnancy was confirmed, rats were housed individually and assigned to two groups: control group (C), with free access to food and water, and undernutrition group (Und), receiving 60% of the average intake of the control group. Standard pellet chow was provided (Nuvital^®^, Nuvilab, Brazil), containing 22% protein, 54% carbohydrates, and 20% fat. Maternal body weight was measured daily throughout pregnancy.

After delivery, all mothers were allowed free access to diet, and the undernourished group was further divided into two subgroups: (Arg) supplemented with an arginine-enriched diet (Impact^®^), and (Hh) supplemented with a hypercaloric-hyperproteic diet (Nutren^®^). Supplementation was administered daily by gavage, with 5 mL of the respective diet. The diets composition is presented in Table 1, and the daily supplementation for rat is presented in Table 2.

**Table 1:** Supplementation diets composition for 100 mL.

	Impact^®^	Nutren^®^
Energy (kcal)	107	106
Carbohydrate (g)	14	7.2
Protein (g)	6.5	7.5
L-arginine (mg)	1,400	–
Fats (g)	2.8	5

Source: Elaborated by the authors.

**Table 2:** Daily supplementation for the rats during lactation period.

	Group Arg	Group Hh
Energy (kcal)	5	5
Carbohydrate (g)	0.7	0.36
Protein (g)	0.325	0.375
L-arginine (mg)	0.7	–
Fats (g)	0.14	0.25

Source: Elaborated by the authors.

During the lactation period, maternal and pup weights were recorded daily. At weaning (day 21 of life), body weight (mothers and pups), as well as body and tail length (pups), was measured. At this stage, all pups underwent a transverse supraumbilical laparotomy followed by abdominal wall closure under anesthesia with ketamine and xylazine. On postoperative day 7, the pups were euthanized by ketamine and xylazine overdose. Approximately 1 mL of blood was collected from the superior vena cava for serum albumin measurement, and abdominal wall healing was assessed by two methods: tensiometry, to determine tensile strength, and histology with picrosirius red staining to quantify type I (mature) and type III (immature) collagen. for the tensiometry analysis, the Emic^®^ model dl-500 instrument was used. A constant traction was applied to both ends of the incision, and the rupture tensile strength was determined. For the collagen studies, the edges of the abdominal wound were resected and stained with picrosirius red. The area stained in red was considered mature collagen (type I), whereas the area stained in green was considered immature collagen (type III). The percentage of each one was determined. The study design is presented in Fig. 1.

**Figure 1:** Study design.

Gestation 21 days (Rats)	Lactation 21 days (Rats)	Weaning 21st days (Pups)	7st posoperative (Pups)
**Control (C)** Ad libitum	**Control (C)** Ad libitum	Transverse laparotomy	Sacrifice
**Undernutrition (Und) -** pair fed	**Arg** Arginine-enriched diet	Transverse laparotomy	Sacrifice
	**Hh** Hypercaloric-hyperproteic diet	Transverse laparotomy	Sacrifice

Source: Elaborated by the authors.

Statistical analysis was performed using Student’s t-test, analysis of variance (ANOVA), and Tukey’s *post hoc* test. *p* <0.05 was considered significant.

## Results

**Figure 2:**
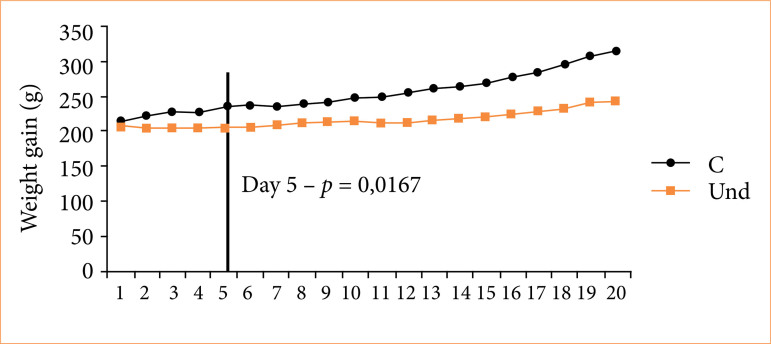
Weight gain during gestation (g).

After a three-day mating period, sperm was detected in the vaginal fluid of 11 out of 20 rats. Three rats were assigned to the control group (C), and eight to the undernutrition group (Und). There was no initial difference in body weight between groups (Table 3), but after five days of undernutrition, study groups showed significant lower body weight (Fig. 2). One undernourished rat failed to deliver, although no bleeding was observed in the cage.

**Table 3:** Body weight at pregnancy detection and at birth in grams.

	Pregnancy	At birth	
		Rats	Pups
Control	206.71 ± 11.84 g	323.86 ± 29.17 g*	8.76 ± 0.89 g*
Undernutrition	205.50 ± 26.89 g	248.92 ± 26.89 g	6.80 ± 1.24 g

* *p* < 0.05.

Source: Elaborated by the authors.

C: control group; Und: undernutrition group. Source: Elaborated by the authors.

**Figure 3:**
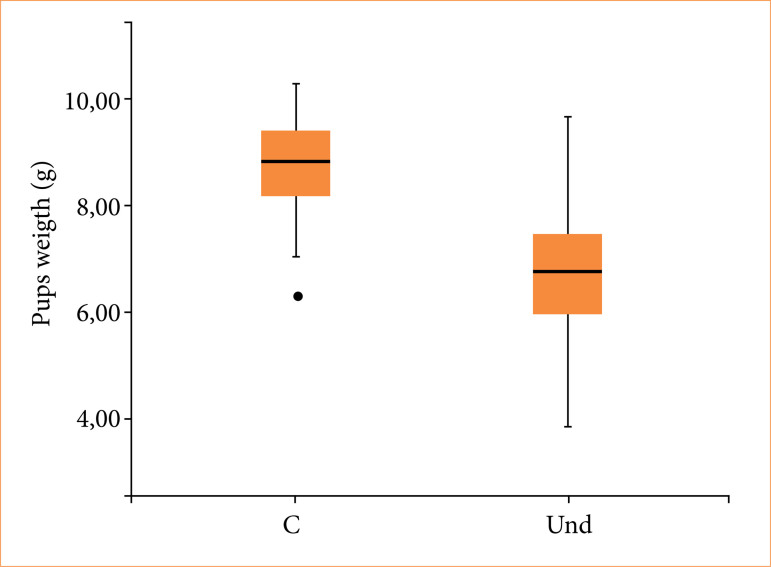
Pups birth weight at birth (g).

At birth, mothers and pups from the undernourished group exhibited significantly lower body weights compared to control group (Table 3 and Fig. 3).

C: control group; Und: undernutrition group; *p* < 0.005. Source: Elaborated by the authors.

After weaning, both supplemented groups (Arg and Hh) exhibited significantly greater body weight and length as compared with the control group (C). No significant difference was observed in tail length. Likewise, there were no significant differences in anthropometric measures between the two supplemented groups (Arg and Hh) (Table 4).

**Table 4:** Pups weight, body and tail length after weaning.

	Weight	Body length	Tail length
Control	29.21 ± 2.86 g*	9.88 ± 0.32 cm*	6.72 ± 0.29 cm
Arginine	36.08 ± 5.38 g	10.91 ± 0.73 cm	7.02 ± 0.59 cm
Hypercaloric	34.66 ± 4.53 g	10.56 ± 0.63 cm	6.93 ± 0.65 cm

* *p* < 0.05 compared to groups arginine and hypercaloric.

Source: Elaborated by the authors.

Postmortem albumin levels were significantly higher in the control group as compared with both supplemented groups (C = 4.55 ± 0.21 g/dL; Arg = 3.75 ± 0.11 g/dL; Hh = 3.62 ± 0.05 g/dL; *p* < 0.05) (Table 5).

**Table 5:** Pups weight and serum albumin measurement at sacrifice.

	Weight	Serum albumin
Control	60.88 ± 1.98 g	4.55 ± 0.21 cm*
Arginine	70.24 ± 2.83 g	3.75 ± 0.11 cm
Hypercaloric	57.01 ± 1.83 g	3.62 ± 0.05 cm

* *p* < 0.05.

Source: Elaborated by the authors.

The first parameter used to evaluate abdominal wall healing, tensiometry, showed that pups from arginine-supplemented dams recovered the tensile strength without significant differences as compared with the control group. In contrast, this recovery was not observed in pups from hypercaloric-supplemented dams (Table 6), which remained significantly lower than controls.

**Table 6:** Pups abdominal wall tensile strength.

	Tensile strength (kgf/cm^2^)
Control	5.4638 ± 0.301
Arginine	6.0763 ± 0.265
Hypercaloric	3.2889 ± 0.314*

* *p* < 0.05.

Source: Elaborated by the authors.

Histological evaluation of the abdominal wall did not reveal significant differences in the proportions of mature (type I) and immature (type III) collagen fibers among the groups (Table 7).

**Table 7:** Pups weight and serum albumin measurement at sacrifice.

	Total collagen	Type I collagen (%)	Type II collagen (%)
Control	4,562.78 ± 601.43	47.04 ± 4.88	52.96 ± 4.88
Arginine	3,189.34 ± 508.36	46.76 ± 4.20	53.23 ± 4.19
Hypercaloric	4,849.43 ± 785.33	38.41 ± 3.97	61.58 ± 3.96

*p > 0.05. Source: Elaborated by the authors.

## Discussion

Recent data indicate that wasting and stunting remain responsible for approximately 2.2 million deaths in children under 5 years of age worldwide each year2,3. Undernutrition may begin as early as prenatal development, and the concept of the “1,000-day window of opportunity” highlights the importance of nutritional interventions during gestation to improve childhood nutritional outcomes4.

Several experimental models have been described to study intrauterine malnutrition, including hypoproteic, hypocaloric, or combined hypoproteic–hypocaloric regimens^17^. In the present study, a pair-fed regimen with 40% restriction was effective in inducing a malnourished state in pregnant rats.

The deleterious effects of prenatal malnutrition have been well documented since 1992, with studies on survivors of the Dutch famine. Barker et al. reported long-term complications involving the cardiovascular system, as well as carbohydrate and lipid metabolism. Based on these findings, multiple studies have demonstrated the negative effects of intrauterine malnutrition on fetal growth and the development of various systems^5-10^.

Coelho-Lemos et al.^17^ described a direct relationship between maternal food restriction and low birth weight. Consistent with this, our study confirmed significant weight differences in undernourished rats after the fifth day of pregnancy. Furthermore, the experiment reproduced the detrimental effects on litter birth weight and length, with significant differences between the control and undernourished groups.

Mateo et al.^31^ proposed maternal arginine supplementation as a strategy to enhance neonatal piglet weight gain. The central concept of their study was to demonstrate the amino acid’s capacity to stimulate breast milk production and improve neonatal growth.

To our knowledge, the present study is the first one to describe an experimental rat model of maternal arginine supplementation. Maternal arginine use proved effective in stimulating weight and length gain in suckling pups. The “arginine–proline cycle between mother and neonate” described by Mateo et al.^31^ proposes that arginine catabolism products act within the mammary gland to improve milk production. Nitric oxide may promote vasodilation and mammary blood flow, while polyamines enhance cellular proliferation and glandular growth^27,28^.

This experiment demonstrated catch-up growth in both weight and length at weaning among pups of rats that were undernourished during pregnancy but received supplementation during lactation. Both supplementation groups, hypercaloric/hyperproteic and arginine-enriched diets, presented significantly greater weight and length than the control group.

Serum albumin levels, a parameter of chronic nutritional status, were assessed one week after weaning. Despite maternal supplementation improving growth recovery, no effect was observed on albumin levels in the pups.

The correlation between adequate nutritional status and efficient wound healing has been extensively documented. Furthermore, immunonutrition through amino acid supplementation has been shown to enhance tissue repair. In this model, pups from arginine-supplemented dams exhibited significant catch-up in wound tensile strength, as well as collagens types I and III concentrations, reaching values comparable to those of the control group.

## Conclusion

Supplementation of arginine in lactating dams that had been undernourished during gestation improved weight and length gain in pups exposed to intrauterine malnutrition and enhanced their wound healing capacity.

## Data availability statement

Data sharing is not applicable.
